# Cardiovascular Magnetic Resonance for the Differentiation of Left Ventricular Hypertrophy

**DOI:** 10.1007/s11897-020-00481-z

**Published:** 2020-08-26

**Authors:** Matthew K. Burrage, Vanessa M. Ferreira

**Affiliations:** University of Oxford Centre for Clinical Magnetic Resonance Research (OCMR), Radcliffe Department of Medicine, University of Oxford, Level 0, John Radcliffe Hospital, Oxford, OX3 9DU UK

**Keywords:** Left ventricular hypertrophy (LVH), Cardiovascular magnetic resonance (CMR), T1 mapping

## Abstract

**Purpose of Review:**

Left ventricular hypertrophy (LVH) is a common presentation encountered in clinical practice with a diverse range of potential aetiologies. Differentiation of pathological from physiological hypertrophy can be challenging but is crucial for further management and prognostication. Cardiovascular magnetic resonance (CMR) with advanced myocardial tissue characterisation is a powerful tool that may help to differentiate these aetiologies in the assessment of LVH.

**Recent Findings:**

The use of CMR for detailed morphological assessment of LVH is well described. More recently, advanced CMR techniques (late gadolinium enhancement, parametric mapping, diffusion tensor imaging, and myocardial strain) have been used. These techniques are highly promising in helping to differentiate key aetiologies of LVH and provide valuable prognostic information.

**Summary:**

Recent advancements in CMR tissue characterisation, such as parametric mapping, in combination with detailed morphological assessment and late gadolinium enhancement, provide a powerful resource that may help assess and differentiate important causes of LVH.

## Introduction

Left ventricular hypertrophy (LVH), defined as an increase in LV mass or wall thickness, is commonly encountered in clinical practice and is associated with structural myocardial changes [1]. It independently predicts adverse cardiovascular outcomes in large population-based studies [2–4].

There are multiple causes of LVH, ranging from physiological adaptation to athletic training or increased afterload (such as hypertension or aortic stenosis), to more severe pathological hypertrophy, as seen in hypertrophic cardiomyopathies and infiltrative/storage diseases. Although clinical and family history, along with physical examination, may narrow the differential diagnosis, the exact aetiology can remain unclear. Given that the management and prognosis of different aetiologies of LVH may differ significantly, an accurate diagnosis is crucial.

Cardiovascular magnetic resonance (CMR) is the current imaging gold standard for accurate and reproducible assessment of cardiac mass, volumes and function [5–7], and is superior to echocardiography (TTE) in the assessment and differentiation of LVH [5, 8, 9]. Its excellent spatial resolution allows evaluation of cardiac structure and function, as well as the presence, symmetry and distribution of hypertrophy [10]. Although the detection of hypertrophy (usually defined as an LV wall thickness ≥ 13 mm) [11] opens a broad differential diagnosis, the presence of increased wall thickness itself rarely establishes the aetiology. Classic imaging features may point to a specific diagnosis (e.g. asymmetrical septal hypertrophy and LV outflow tract obstruction in hypertrophic cardiomyopathy), but these are not always sensitive or specific. Pathological hypertrophy is more likely associated with other changes in myocardial tissue or function, such as myocardial fibrosis, myofibrillar disarray, subclinical dysfunction or abnormal protein deposition, all of which may be detectable non-invasively by advanced CMR tissue characterisation techniques.

## CMR Tissue Characterisation Techniques

### Late Gadolinium Enhancement

Late gadolinium enhancement (LGE) is the standard CMR myocardial tissue characterisation technique. It allows differentiation of ischaemic and non-ischaemic heart disease via characteristic enhancement patterns, and is excellent for detecting areas of focal scarring/fibrosis [12]. LGE is more likely to be associated with pathological hypertrophy, and thus plays a key role in the differentiation of LVH. However, to highlight areas of pathology, it relies on areas of presumed normal myocardium for nulling. Detection of diffuse myocardial fibrosis may thus be challenging, particularly if gadolinium-based contrast agent (GBCA) uptake is uniform. Other complementary tissue characterisation techniques can address this limitation.

### Parametric Mapping

Novel parametric mapping techniques, such as T1 and T2 mapping, allow advanced tissue characterisation via directly quantitative pixel-wise maps [13]. Detailed review of the technical aspects and MR physics principles of parametric mapping may be found elsewhere [14••]. Native (pre-contrast) T1 values are sensitive to increased tissue free-water content and are prolonged by myocardial inflammation and oedema, as well as areas of focal and diffuse fibrosis [15]. Conversely, T1 values may be lowered by high tissue iron content, lipid deposition (as seen in Fabry disease) or GBCAs [16–18]. T1 mapping is well validated in detecting subtle myocardial changes in the early stages of a wide range of myocardial disease [15, 19–22], and is especially useful in the differentiation of LVH [14••].

Similar to native T1 mapping, T2 mapping also reflects global signal from the intra- and extra-cellular myocardial compartments. An elevated T2 generally indicates increased free-water content and is typically used to detect acute myocardial inflammation and oedema [14••]. Although less relevant in the routine assessment of myocardial hypertrophy, there may be incremental value of T2 mapping in specific instances; cases of acute myocardial oedema and increased wall thickness following acute myocardial injury have been reported [23], while increased T2 signals have been observed in Fabry’s disease, suggesting an inflammatory component in its pathophysiology [24].

Additionally, there is increasing interest to quantify the myocardial extracellular volume (ECV), which may act as a surrogate marker for diffuse interstitial fibrosis after exclusion of confounding factors [25–27]. ECV is calculated using pre- and post-contrast myocardial and blood T1 values with adjustment for blood haematocrit. An expanded ECV has been noted in hypertensive heart disease with LVH, and associated with adverse outcomes in large patient cohorts [14••, 28•].

### Contractility and Myofibre Assessments

Other advanced CMR techniques to assess LVH include methods to evaluate myocardial contractility and pathological myofibre disarray. Myocardial deformation (strain) parameters, such as global longitudinal strain (GLS) and global circumferential strain (GCS), have emerged as sensitive markers of early subclinical myocardial dysfunction. They are strong, independent predictors of mortality in patients with heart disease, even after accounting for left ventricular ejection fraction (LVEF) and LGE burden [29]. Feature tracking (CMR-FT), which tracks myocardial borders over time on cine images, is well validated and evaluated in a wide range of cardiovascular disease [30, 31], including the differentiation of LVH [32].

Diffusion tensor imaging (DTI) is an emerging CMR technique that may provide important insights into hypertrophic disease processes by characterising myocardial microstructural changes [33]. It can assess cardiomyocyte fibre orientation and packing (and hence myofibrillar disarray), by mapping the three-dimensional diffusion of water molecules [33, 34, 35•]. The ability to detect microstructural changes in hypertrophic diseases may help differentiate pathological from physiological hypertrophy (expected to have normal myocardial architecture). Although currently limited outside research centres, it has potential to progress to a clinically useful diagnostic and prognostic tool.

**Fig. 1:**
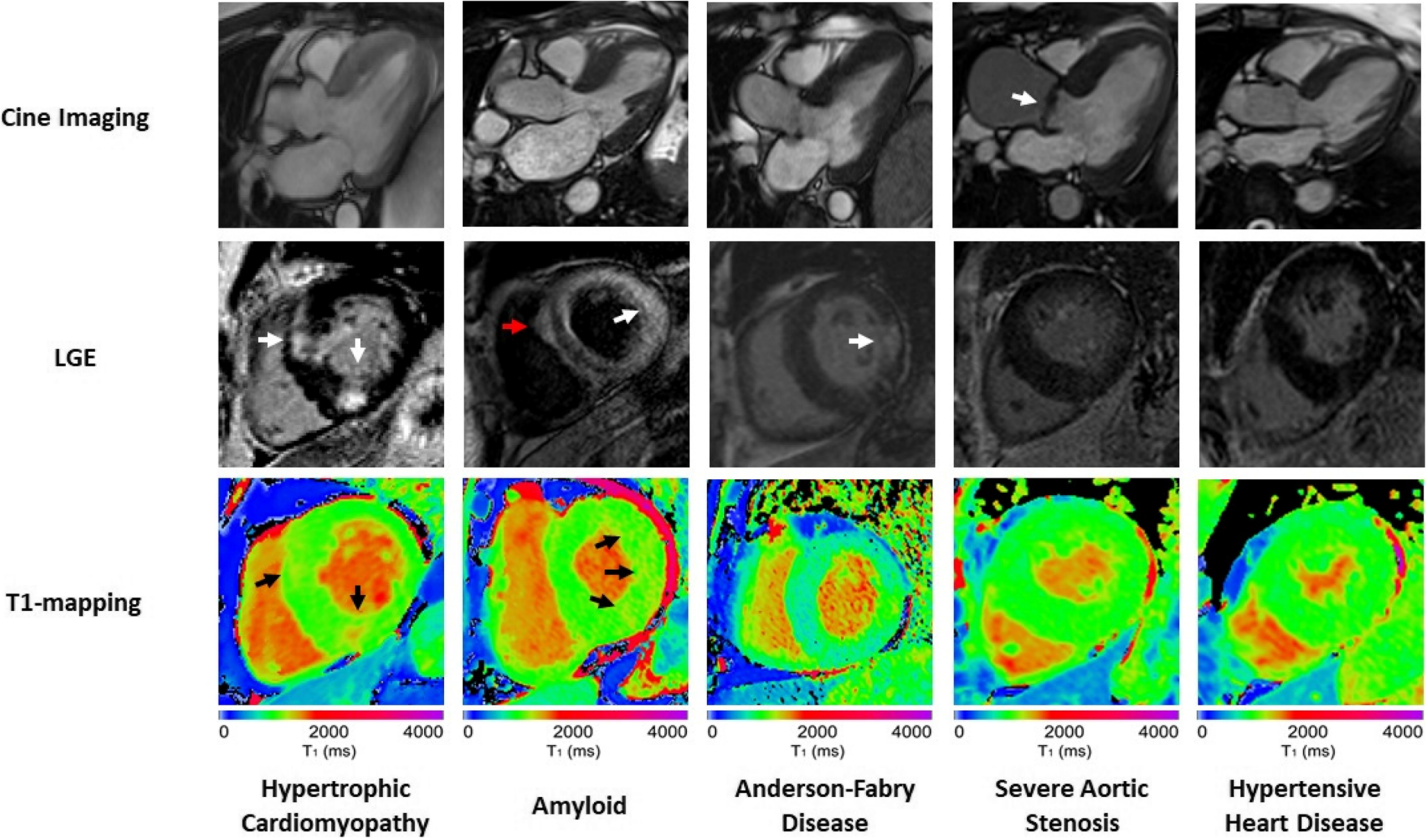
Differences in anatomical and tissue characterisation features on CMR between LVH phenotypes. Although similar appearances on long-axis anatomical cine imaging, LGE and T1 mapping were able to differentiate between phenotypes in most cases. The HCM case demonstrates insertion point LGE (arrows) as well as minor diffuse enhancement in the septum with corresponding red patches of fibrosis on T1 maps (arrows). The amyloid case demonstrates abnormal LGE kinetics with biventricular subendocardial septal enhancement in the classic ‘zebra’ pattern in the septum (red arrow), along with diffuse myocardial uptake elsewhere (white arrow), and significantly elevated native T1 values > 1100 ms (normal range 941 ± 23 ms at 1.5 T), denoted by abnormal red patches (arrows) on T1 mapping. The AFD case demonstrates posterior wall scar on LGE (arrow) and characteristically low native T1 values < 800 ms, denoted by the patchy blue appearance to the myocardium (with an area of T1 pseudonormalisation corresponding to the posterior wall scar). The aortic valve is severely calcified and restricted (arrow) in the severe aortic stenosis case, with no LGE but T1 values of 982 ms approaching the upper limits of normal. The HHD case presents a milder hypertrophic phenotype with no significant abnormalities on LGE or T1 mapping.

These powerful CMR techniques play an important role in differentiating pathological and physiological hypertrophy. The next section will focus on their application in the most commonly encountered LVH phenotypes, namely hypertrophic cardiomyopathy (HCM), infiltrative/storage diseases (cardiac amyloidosis, Anderson-Fabry disease [AFD]), increased afterload (hypertensive heart disease, aortic stenosis) and physiological remodelling in the athletic heart. Key differentiating CMR characteristics are displayed in Fig. 1 and Table 1.

**Table 1:** Imaging characteristics of different LVH phenotypes

	Morphology	LGE	T1	ECV
HCM	LVH > 15 mm in 1 or more myocardial segments (in absence of abnormal loading conditions) Pattern of LVH (asymmetric septal hypertrophy most common; concentric, focal, and apical variants also exist) Myocardial crypts may be present	Present in 50–65% of cases Focal at RV/LV insertion points + diffuse hazy enhancement in areas of maximal hypertrophy	↑	↑
Amyloid	Concentric LVH and RVH with small cavity Biatrial dilatation Thickened subvalvular apparatus Thickened interatrial septum Pericardial effusion	Abnormal gadolinium kinetics with poor myocardial nulling and high myocardial uptake Global subendocardial LGE most commonly described (tram-line pattern)	↑↑↑	↑↑↑
AFD	Pattern of LVH (typically concentric; asymmetric septal hypertrophy and RVH may also occur)	Basal inferolateral midwall scar (~ 50% of cases)	↓↓	↔
HHD	Concentric LVH (typically < 15 mm)	Non-specific, midwall enhancement	↔/↑	↔/↑
AS	Concentric LVH (asymmetric patterns also described)	Non-ischaemic midwall fibrosis	↑	↑
Athlete’s heart	Concentric LVH (typically < 13-16 mm) LV cavity dilatation (> 54 mm)	Typically absent	↔	↔/↓

## Hypertrophic Cardiomyopathy

Hypertrophic cardiomyopathy (HCM) is the most common genetic heart muscle disease (prevalence ~ 1:500 to 1:200) and the leading cause of sudden cardiac death (SCD) in the young [36]. It is defined by increased left ventricular wall thickness (≥ 15 mm) in one or more myocardial segments not solely explained by abnormal loading conditions (such as hypertension or aortic stenosis) [37]. Differentiating HCM from other common hypertrophic phenotypes can be challenging, particularly in milder forms of the disease which may have considerable overlap. Although clinical history may establish a familial link (typically an autosomal dominant inheritance pattern due to mutations in cardiac sarcomere genes), advanced cardiac imaging is often needed to further differentiate.

CMR has a Class I recommendation in current HCM guidelines by both the European Society of Cardiology (ESC) and American College of Cardiology (ACC) if echocardiography is insufficient, if there is ongoing diagnostic uncertainty regarding hypertrophic phenotypes or if additional information is required [37, 38]. Although TTE provides important information on ventricular function and morphology (in patients with good acoustic windows), CMR can more precisely characterise the location, distribution and extent of LVH. It is particularly helpful in diagnosing HCM in patients with poor echo windows, or when some regions are poorly visualised, such as the cardiac apex, very basal segments, lateral walls and right ventricle. Myocardial crypts, associated with (although not specific for) HCM, may be better seen on CMR. However, it is the powerful tissue characterisation capabilities of CMR which provides the most added value over echocardiography. Identification of potentially pro-arrhythmic substrates, like myocardial fibrosis or myofibrillar disarray, has been proposed to stratify individual patient risk.

### Tissue Characterisation in HCM

#### Late Gadolinium Enhancement

The presence and pattern of LGE is particularly useful for diagnosis and risk stratification in HCM. LGE has been shown to correlate with, although not specific for, areas of increased myocardial collagen and fibrosis on histological analysis of myectomy specimens and explanted hearts [39–41]. A commonly described pattern is focal enhancement at the RV insertion points (anterior, inferior or both), along with diffuse patchy or hazy mid-wall enhancement in areas of hypertrophy [42]. Approximately 50–65% of HCM patients will demonstrate LGE [37, 38, 43]. Its presence makes physiologic or athletic remodelling unlikely and is more suggestive of underlying pathology [43, 44]. The presence and extent of LGE is also independently associated with an increased risk of SCD and adverse outcomes (including development of heart failure), particularly if > 15% of total LV mass [43, 45•, 46–48]. Although not yet incorporated into risk calculators, it is clear that LGE in HCM is prognostic and may even outperform and increase the discriminative power of current SCD risk scores [49].

#### T1 Mapping and ECV

Advances in T1 mapping and ECV quantification have allowed non-invasive CMR assessment of diffuse myocardial fibrosis or other changes that may be missed by LGE. In patients with dilated cardiomyopathy (DCM) or HCM, even segments with normal wall thickness and no LGE may have increased T1 values, suggesting underlying disease processes beyond those assessed by LGE [50]. Furthermore, an elevated ECV has been seen in genotype-positive HCM patients, both in the presence and absence of LVH, compared with controls [51]. Native T1 mapping and ECV have been successful in differentiating clear-cut HCM from hypertensive heart disease and normal controls on a group level, and may also be useful in screening genotype-positive phenotype-negative subjects [52]. Radiomic texture analysis of native T1 images has recently discriminated between hypertensive heart disease and HCM patients, providing incremental value over average native T1 values alone [53•]. These novel parametric mapping techniques, when used in combination with morphology assessments and LGE, may aid identification of areas of myocardial abnormalities more likely indicative of HCM when the diagnosis is otherwise not clear-cut. Further work is required to validate these methods for the reliable differentiation of HCM from other causes of LVH in individual patients.

#### Strain and Diffusion Tensor Imaging

Myocardial strain imaging may also help differentiate between pathological and physiologic hypertrophy. One recent study reported that impaired CMR-FT GLS may differentiate between HCM and hypertensive heart disease, with a similar (although slightly inferior) discriminatory capacity to tissue characterisation (LGE, parametric mapping) biomarkers [32]. However, other studies have found that GLS is unable to differentiate between these phenotypes in cases with maximal LV wall thickness ≥ 15 mm [54]. Further work is required to conclusively establish the utility of strain imaging in the differentiation of LVH aetiologies and severity of disease, whether alone or in combination with other biomarkers.

Finally, diffusion tensor imaging (DTI) is an important recent CMR advancement for diagnosis and risk stratification in HCM with good reproducibility [34, 55, 56]. It has prognostic implications, with a recent study showing that increased myofibrillar disarray in HCM patients was associated with ventricular arrhythmias, even after correcting for conventional risk factors [35•]. Although availability is currently limited, DTI is nonetheless promising for diagnosis and risk stratification in HCM in its ability to identify pathological microstructural myocardial changes, which may not be evident in more benign causes of LVH.

## Cardiac Amyloidosis

Cardiac amyloidosis is a rare systemic disease characterised by progressive myocardial infiltration of misfolded protein fibrils. The resulting phenotype is an infiltrative cardiomyopathy with increased myocardial wall thickness, conduction disease and subsequent heart failure. Although often mislabelled as hypertrophy, the increased wall thickness is due to extracellular expansion from infiltration rather than true cardiomyocyte hypertrophy. Two main forms of amyloid affect the heart: light chain (AL) amyloidosis, where amyloid fibrils are derived from monoclonal immunoglobulin light chains in association with a plasma cell dyscrasia [57], and transthyretin amyloidosis (ATTR). ATTR amyloid cardiomyopathy results from the accumulation of either wild-type (ATTRwt) or hereditary/mutated (hATTR) transthyretin protein [58]. Correct identification of the amyloid subtype (i.e. AL or ATTR) is essential, due to differing clinical courses and treatments.

AL amyloidosis generally takes a more fulminant course and has a poor prognosis; untreated, the median survival from onset of heart failure may be approximately 6 months [59]. More severe heart failure may be seen, possibly due to direct myocardial light chain toxicity in addition to infiltration [57]. However, AL amyloid is responsive to chemotherapy, and indeed, modern therapies may induce a prolonged remission, provided it is diagnosed and treated early.

ATTR amyloidosis has a more indolent and slowly progressive clinical course, with median survival approximately 60 months from first heart failure presentation [60]. Until recently, there were no effective treatments for ATTR amyloid cardiomyopathy beyond symptom relief. A new transthyretin protein stabiliser (Tafamidis) has improved all-cause mortality, reduced cardiovascular-related hospitalisations and reduced declines in functional capacity and quality of life [61••]. Despite differences in progression, treatments and clinical outcomes between AL and ATTR amyloid, it is the degree of myocardial amyloid involvement that determines prognosis. Early diagnosis is thus critical to allow effective treatment.

Morphologically, cardiac amyloidosis is characterised by increased biventricular wall thickening (typically concentrically with a small LV cavity), thickening of the subvalvular apparatus and atrial septum, biatrial dilatation and pericardial effusion. LVEF is generally preserved until the end stages of disease. Echocardiography is the most used diagnostic imaging test, with the characteristic apical sparing pattern on strain imaging often helping to differentiate amyloid from other causes of LVH [62]. However, morphological characteristics are not specific and are often ascribed to other more common hypertrophic diseases.

Increasingly sensitive advanced cardiac imaging techniques, such as technetium-labelled bone scintigraphy (e.g. 99mTc-DPD scans) and CMR, have led to greater recognition and earlier detection of cardiac amyloid. DPD scans [63, 64] in particular have proven very effective at diagnosing cardiac amyloid, and have excellent sensitivity and specificity when working within a proposed diagnostic framework [65, 66]. They have led to a new diagnosis of amyloid in 13% of patients with heart failure with preserved ejection fraction [67] and 16% of patients undergoing transcatheter aortic valve replacement [68], consistent with rates seen at autopsy [69]. However, DPD scans require ionising radiation and radioactive tracers and are unable to differentiate between other non-amyloid causes of LVH.

CMR tissue characterisation provides a radiation-free alternative to differentiate cardiac amyloid from other causes of LVH. Characteristic LGE patterns are seen; myocardial and blood-pool gadolinium kinetics are abnormal [70], and the blood-pool is often atypically dark, reflecting high myocardial uptake and fast washout [71]. There may be such extensive interstitial expansion that the myocardial extracellular volume mirrors the blood plasma volume. Myocardial nulling is poor due to the lack of relative normal myocardium. Global subendocardial LGE is most commonly described, with a ‘tram-line’ or ‘zebra’ pattern [42], although patchy, diffuse or transmural enhancement may also occur.

Parametric T1 mapping plays a prominent role and offers potentially improved sensitivity for the early detection of amyloid compared with LGE [72]. Native T1 values are significantly elevated in cardiac amyloid, typically much higher than in other diffuse or hypertrophic diseases [14••, 72, 73]. ECV has been used as a surrogate marker for amyloid burden (amyloid typically has the highest ECV of all cardiomyopathies), and carries important prognostic value [74, 75]. T1 mapping and ECV may also be used to track therapeutic response [76, 77].

Like echocardiography, CMR-FT studies have shown the presence of marked relative apical sparing of longitudinal strain patterns in patients with cardiac amyloid. One study showed that regional reductions in longitudinal strain helped to differentiate cardiac amyloidosis from other hypertrophic mimics, such as HCM and AFD, with a base-to-apex quantitative gradient of LGE burden also identified [78]. Myofibrillar disarray on DTI was seen in one study, with excellent correlations with native T1 and ECV measures [79•]. Interestingly, myofibres were seen to exist in a more circumferential orientation in cardiac amyloid patients compared with healthy controls, which may provide a rationale for the classically described reductions in longitudinal strain [79•].

## Anderson-Fabry Disease

Anderson-Fabry disease (AFD) is an X-linked inherited metabolic disease caused by the reduction or absence of a functional α-galactosidase A enzyme. This results in lysosomal accumulation of glycosphingolipids in many organs and tissues [80]. The clinical syndrome is often one of progressive renal, cardiac and cerebrovascular disease. Cardiac involvement is characterised by progressive LVH (due to intracellular accumulation of sphingolipids), myocardial inflammation and fibrosis, conduction disease, arrhythmias, valve dysfunction and heart failure. Although rare (the estimated general prevalence of AFD is 1 in 40,000 to 170,000) [80, 81], AFD has the potential to be reversed or stabilised with recombinant enzyme replacement therapy [82, 83]. One of the key indications to start enzyme replacement therapy is the absence of myocardial fibrosis in patients with LVH (> 12 mm) and other clinical signs and symptoms [84]. Differentiating AFD from other hypertrophic mimics and determining the extent of myocardial fibrosis are thus crucial for treatment decision-making and prognostication.

Morphological imaging assessment may display characteristic hypertrophic features but is not sufficient alone to diagnose AFD. For example, although the pattern of LVH is usually concentric, RV hypertrophy, asymmetric septal hypertrophy and even LVOT obstruction may occur, indistinguishable from that seen in sarcomeric HCM [85]. Tissue characterisation is crucial for further differentiation. The characteristic CMR-LGE pattern of basal inferolateral mid-wall scar is seen in ~ 50% of cases [86]. However, other cardiac conditions (e.g. myocarditis, desmosomal disease) may cause similar posterior wall fibrosis; more advanced imaging differentiation is needed.

T1 mapping plays a prominent role in the diagnosis of AFD [14••]. Significant lipid infiltration shortens myocardial T1 relaxation times, and it is not surprising that AFD, which is associated with intracellular sphingolipid accumulation, has significantly lower T1 values compared with other causes of LVH [18]. It is important to recognise, however, that T1 values may also be paradoxically elevated when a tissue voxel is only partially occupied by fat, seen in commonly used T1 mapping methods that are based on balanced steady state free precession [87]. Nonetheless, T1 mapping seems to differentiate AFD from other hypertrophic diseases, without significant overlap and independent of hypertrophy and sex [18, 88]. There may be an inflammatory component to the AFD cardiomyopathy, with elevated T2 values seen particularly in areas of late gadolinium enhancement, which are not present in patients with HCM or chronic myocardial infarction [24, 89]. Segmental pseudonormalisation or elevation of T1 in the basal inferolateral wall may also occur, likely reflecting areas of mixed storage and fibrosis or inflammation [18].

Myocardial strain imaging has also been investigated in AFD. Impaired longitudinal strain correlates with reduced native T1 values and increased LV wall thickness and mass [90]. Loss of the base-to-apex circumferential strain gradient may represent an early (pre-LVH) marker of cardiac involvement in AFD [91]. Although they may provide useful additional information, strain measurements in isolation currently lack the power to differentiate between hypertrophic phenotypes. One study has shown that the combination of LGE and strain could differentiate cardiac amyloid from HCM and AFD, but was not powered for further comparisons; further research is required [78].

Other lysosomal and glycogen storage disorders (e.g. Danon disease, Pompe disease) are also classically associated with LVH. These conditions are often progressive and may manifest extreme hypertrophic phenotypes. Given their rarity, there is little data available on using advanced cardiac imaging to differentiate them from other causes of LVH [92, 93]. For now, genetic testing, particularly in the presence of clinical ‘red flags’, provides the greatest diagnostic and prognostic information for these rarer presentations [94].

## Pressure-Loaded Hypertrophy (Hypertensive Heart Disease and Aortic Stenosis)

Hypertensive heart disease (HHD) and aortic stenosis are common conditions which cause adaptive LVH due to increased afterload. Systemic hypertension is one of the most common chronic diseases, and is a well-established cardiovascular risk factor [95]. In hypertensive patients, the incidence of LVH is generally related to the level of systolic blood pressure control, although other factors, such as age, sex, race and body mass index, are also influential. Although a careful clinical history and examination is often sufficient to diagnose HHD, myocardial imaging may be required to help differentiate or exclude co-existent pathology, such as amyloidosis or HCM.

LVH secondary to HHD is more likely to regress with anti-hypertensive treatment on serial imaging (in contrast to hypertrophic or infiltrative cardiomyopathies), but this is not particularly sensitive [37]. Maximal LV wall thickness can help discriminate—it is significantly greater in patients with unequivocal HCM or pathological hypertrophy—but there may be considerable overlap [96]. The majority of hypertensive patients with LVH have a maximal LV wall thickness < 15 mm, but some demographics (particularly in the presence of chronic kidney disease) may have wall thickness up to 20 mm [97]. The pattern of hypertrophy may also be useful; LVH secondary to HHD is typically concentric, while the presence of focal LVH, apical or RV involvement favours a diagnosis of HCM. LGE has been reported in HHD in up to 50% of patients in a non-specific, non-subendocardial pattern [98], although generally to a lesser extent than is seen in HCM. T1 mapping and ECV values are slightly increased in hypertensive patients with LVH, but overall, these changes are small [99]. One study showed that CMR-FT GLS was able to differentiate between HHD and HCM, although the overall discriminatory capacity was similar (and indeed slightly inferior) to CMR tissue characterisation biomarkers, such as LGE and parametric mapping [32]. HHD remains a challenging scenario for the clinician, and a definitive diagnosis cannot always be made without multiple testing, longitudinal follow-up and re-assessment to treatment response to exclude other causes of LVH.

The diagnosis of significant aortic stenosis (AS) is generally apparent from clinical history, examination and echocardiography. However, there may be considerable variation in the LV hypertrophic response, with the pattern and severity of LV remodelling not always correlating with the degree of valve narrowing [100]. Asymmetric patterns of LVH have been described [100], and may have significant overlap in appearance with HCM. Tissue characterisation, in addition to morphological assessment, may have better yield in terms of both diagnosis and prognostication.

Myocardial fibrosis occurs as part of the hypertrophic response to increased afterload, and is a key pathological component of the transition to heart failure and adverse events in AS. Non-ischaemic mid-wall fibrosis seen on LGE is well described as an early marker of LV decompensation and predictor of adverse cardiovascular outcomes, and does not appear to regress with valve replacement [101–104].

Native T1 values increase with worsening severity of aortic stenosis and symptom status, and correlate significantly with histological fibrosis and collagen volume fraction on myocardial biopsy [105]. It is important to recognise, however, that the natural physiological response to the increased demands of the hypertrophied and pressure-loaded ventricle is a compensatory increase in resting coronary blood flow, microvascular dilatation and increased myocardial blood volume [106]. This can also lead to an elevated resting myocardial T1 (due to increased myocardial free water content).

Interrogation of these T1 signal changes using a vasodilator stress agent in severe AS patients shows a ceiling of stress T1 reactivity similar to healthy volunteers, with a blunted overall delta T1 response (between stress and rest, due to increased resting T1 values) [106]. Normalisation of both resting myocardial T1 as well as the vasodilator delta T1 response after aortic valve replacement suggests that the resting T1 changes reflect the effect of severe AS on the vascular compartment, rather than what is often quoted as ‘diffuse fibrosis’ [27, 106].

This principle should be remembered when looking at post-contrast T1 mapping and ECV. An elevated ECV correlates with markers of LV decompensation in severe AS [107, 108], while a recent large multi-centre study showed that an increasing ECV is an independent marker of cardiovascular and all-cause mortality in aortic stenosis, even after adjustment for LGE [109•]. Associations between parametric mapping findings and clinical outcomes need to be interpreted with the underlying pathophysiological disease processes in mind, as they may reflect a worse disease state with loss of vascular reserve rather than true diffuse fibrosis. Nonetheless, tissue characterisation on CMR provides valuable prognostic information in the assessment of patients with aortic stenosis, although differentiating concomitant LVH phenotypes, such as amyloidosis [110], in the presence of severe AS remains challenging.

## Athlete’s Heart

Physiological adaptation to regular intense physical training may result in enlarged cardiac chamber size and increased LV wall thickness and mass [37]. There is a spectrum of overlapping phenotypes, ranging from DCM- to HCM-like morphologies. Reliable differentiation between pathological and physical hypertrophy is crucial, due to the implications for individual athletes and their families [37]. There is no single ‘gold standard’ diagnostic test to differentiate these conditions, although there may be important clinical clues on family history, ECG, morphological imaging and physiologic assessment.

Approximately 2% of white athletes and up to 13% of black athletes have an LV wall thickness of 13–16 mm (thought to be the upper limit of normal for athletic training) [111], which may overlap with a mild phenotype of HCM [44]. In athletes with ‘grey zone’ LVH, other morphological characteristics may help differentiate HCM from athletic training. An unusual pattern of LVH, a small LV cavity with an end-diastolic diameter < 45 mm, abnormal ECG traces and systolic anterior motion of the mitral valve apparatus may all suggest a diagnosis of HCM [112]. On the other hand, an increased LV cavity size (end-diastolic diameter > 54 mm) is rare in HCM (usually only occurring in end-stage heart failure) and is more suggestive of athletic training [113, 114].

CMR tissue characterisation and assessment of myocardial fibrosis are helpful discriminators between physiologic and pathological LVH, with typical LGE patterns more suggestive of HCM. However, absence of LGE cannot exclude HCM, given its presence in only 50–65% of cases. Parametric mapping may also be useful: one study showed normal T1, T2 and ECV values in athletes with increased LV mass, reduced LVEF and increased LV volumes [115]. Another study showed an inverse relationship between ECV and LV wall thickness in athletes, in contrast to direct correlation with pathological HCM, suggesting that physiological LVH results from cardiomyocyte hypertrophy rather than increased extracellular matrix [116]. Further validation of these novel mapping techniques is required before they can be reliably used in this setting.

Exercise testing may provide additional diagnostic and prognostic information, with exercise-induced arrhythmias, an abnormal blood pressure response and an inducible LVOT gradient, all features of HCM which may be unmasked with stress [117]. Cardiopulmonary exercise testing (CPET) may also be useful, with one study showing that elite athletes with LVH had significantly greater maximal oxygen consumption (VO_2_ max) than patients with HCM [118]. However, athletes with concomitant HCM have also been shown to demonstrate normal or supra-normal measures of exercise capacity [114]. Exercise capacity should be interpreted in the context of athletic status, rather than as a sole discriminator. Exercise-induced reductions in myocardial energetics on magnetic resonance spectroscopy in HCM patients provides mechanistic insight into these processes, but further work is required before it can be applied to differentiating LVH phenotypes from athletic remodelling [119, 120]. Measures of cardiac output and contractile reserve on exercise testing are more relevant in differentiating DCM rather than HCM phenotypes, and are reviewed elsewhere [117]. If there is ongoing doubt over the diagnosis, then either genetic testing or a 3-month trial of deconditioning may be considered; regression of LV wall thickness with de-training supports a diagnosis of athlete’s heart [121].

## Future Directions

There is increasing clinical interest to improve the non-invasive phenotyping of cardiovascular diseases down to the tissue and cellular levels, and at faster speed. Recent advances in metabolic imaging using phosphorus magnetic resonance spectroscopy at ultra-high field strengths (e.g. 7-T) and hyperpolarized MRI have allowed unprecedented new insights into physiological and pathological changes in cardiac metabolism [122, 123••]. There is clear potential to further characterise and quantify metabolic changes in hypertrophic disease phenotypes, including at higher resolution [124]. As clinical evidence behind MR tissue characterisation increases, it is expected that they will eventually become part of routine clinical practice, particularly in the assessment of LVH and other cardiomyopathies. Some, like parametric mapping (widely regarded as the fourth era of myocardial CMR development) [13], have already made this transition to clinical practice, with promise to provide functional and tissue characterisation without the need for contrast agents.

Artificial intelligence (AI) and machine learning (ML) approaches are growing rapidly in the field of MRI and are expected to change clinical practice, with particular promise in improving both image acquisition and post-processing times. Automated ML analysis using neural networks has recently been shown to achieve similar precision to human experts in CMR image processing, but at a fraction of the time cost [125•]. This may also be applied to advanced tissue characterisation, with novel radiomics and texture analysis techniques demonstrating significant potential to identify pathological hypertrophic phenotypes beyond that which can be appreciated visually by human experts [126].

Finally, there is active research into ‘fast’ MRI scans, with the expectation that this will translate into improved cost-effectiveness. Fast multi-slice sequences yield excellent LGE image quality for the assessment of myocardial fibrosis at significantly reduced scan time compared with conventional methods [127], while motion-corrected techniques continue to strive to improve acquisition efficiency for parametric mapping without compromising diagnostic accuracy [128]. Compressed sensing (CS), which enables image reconstruction from sparse data, has multiple CMR applications, and may soon become practical for clinical translation due to improvements in computer hardware [129, 130•]. These include single breath-hold three-dimensional whole-heart reconstructions [131], and real-time ‘leadless’ imaging without the need for ECG gating or breath-holding [132]. Further research is needed, but early signs are promising that these technological advances will enable rapid assessments of LVH and cardiac phenotyping in routine clinical applications in the future.

## Conclusion

Identification of the underlying aetiology of LVH remains a challenging but important clinical problem, with significant therapeutic and prognostic implications. Recent novel CMR advances such as parametric mapping, DTI and strain, in combination with detailed morphological imaging and late gadolinium enhancement, have greatly improved our ability to non-invasively and comprehensively evaluate LVH phenotypes. CMR provides important guidance on diagnosis, therapy and prognosis, and is recommended for the differentiation and clinical assessment of patients with LVH.
